# microRNAs as a New Mechanism Regulating Adipose Tissue Inflammation in Obesity and as a Novel Therapeutic Strategy in the Metabolic Syndrome

**DOI:** 10.1155/2014/987285

**Published:** 2014-03-24

**Authors:** Qian Ge, Sonia Brichard, Xu Yi, QiFu Li

**Affiliations:** ^1^Department of Endocrinology, The First Affiliated Hospital of Chongqing Medical University, Chongqing 400016, China; ^2^Endocrinology, Diabetes and Nutrition Unit, Institute of Experimental and Clinical Research, Medical Sector, University of Louvain, 1200 Brussels, Belgium; ^3^Neurology Unit, Daping Hospital, Research Institute of Surgery, The Third Military Medical University, Chongqing 400042, China

## Abstract

Obesity is associated closely with the metabolic syndrome (MS). It is well known that obesity-induced chronic inflammation plays a fundamental role in the pathogenesis of MS. White adipose tissue (AT) is the primary site for the initiation and exacerbation of obesity-associated inflammation. Exploring the mechanisms of white AT inflammation and resetting the immunological balance in white AT could be crucial for the management of MS. Several prominent molecular mechanisms have been proposed to mediate inflammation in white AT, including hypoxia, endoplasmic reticulum stress, lipotoxicity, and metabolic endotoxemia. Recently, a growing body of evidence supports the role of miRNAs as a new important inflammatory mediator by regulating both the adaptive and innate immunity. This review will focus on the implication of miRNAs in white AT inflammation in obesity, and will also highlight the potential of miRNAs as targets for therapeutic intervention in MS as well as the challenges lying in miRNA-targeting therapeutics.

## 1. Introduction

The incidence of obesity defined as body mass index (BMI) ≥ 30 kg/m^2^ has increased drastically worldwide during recent decades. Obesity is associated with a cluster of metabolic disorders, including increased risk of insulin resistance, type 2 diabetes (T2DM), hypertension, dyslipidemia, and cardiovascular disease; these disorders and obesity* per se* constitute a serious threat, known as the metabolic syndrome (MS) [1]. It is well known that chronic inflammation is a key feature of obesity; this obesity-induced inflammation builds the common soil for the pathogenesis of MS [2]. Therefore, resetting the immunological balance in obesity is a crucial approach for the management of MS.

White adipose tissue (AT) has been regarded as the primary goal of pharmacological intervention since obesity-induced inflammation is mainly initiated and exacerbated in this organ [3–5]. Although several prominent molecular mechanisms have been proposed to trigger inflammation in white AT, including hypoxia, endoplasmic reticulum stress, lipotoxicity, and metabolic endotoxemia [5], these factors cannot fully explain the origin of inflammation. On the other hand, current anti-inflammatory strategies are not sufficient enough in the treatment of MS. These facts suggest that there are lots of unknowns responsible for AT inflammation in obesity. In recent years, a growing body of evidence has highlighted the modulating roles of miRNAs in immune/inflammatory system [6, 7] and their involvement in the obesity-related metabolic disorders including T2DM and atherosclerosis [8, 9]. Specific miRNAs have been implicated in adipogenesis and mature adipocyte function [10]. As yet, whether these miRNAs could be another mechanism for mediating AT inflammation and whether these tiny molecules could serve as potent therapeutic anti-inflammatory targets for MS are still not fully settled. The aim of the present review is to address these questions.

## 2. Obesity, Inflammation, and Metabolic Syndrome

### 2.1. Low-Grade Inflammation as a Key Feature of Obesity

During the past decade, it became clear that inflammation is a key feature of obesity [2]. The inflammatory response triggered by obesity involves many components of the classical inflammatory response to pathogens and includes the increases in circulating inflammatory cytokines and acute-phase proteins (e.g., C-reactive protein), recruitment of leukocytes to inflamed tissues, activation of tissue leukocytes, and generation of reparative tissue responses (e.g., fibrosis). However, the nature of obesity-induced inflammation, referred to as “metainflammation” (metabolically triggered inflammation), is unique compared with other inflammatory paradigms (e.g., infection and autoimmune disease) in several key aspects. First, the chronic nature of obesity produces a low-grade activation of the innate immune system that affects metabolic homeostasis over time. Second, childhood obesity may place individuals at risk for lifelong inflammation, since inflammatory markers are elevated in obese children as young as 3 years old. Third, this chronic inflammation is composed of recurrent acute episodes of nutrition-related immune activation induced by nutrient availability (fasting or high-fat meals). This fluctuation may be associated with the induction of pro- or anti-inflammatory mediators. Finally, obesity-induced chronic inflammation is unique by its multiorgan involvement [11].

### 2.2. Obesity-Induced Inflammation Linking Obesity to Metabolic Syndrome

The metabolic syndrome (MS) refers to the clustering of cardiovascular risk factors that include central obesity, hyperglycemia, dyslipidemia, and hypertension. The ultimate importance of this cluster is to identify individuals at high risk of both T2DM and cardiovascular disease. Growing evidence implicates obesity-induced inflammation as an important mechanism linking obesity to the MS in metabolically active organs. Assessment of gene expression networks in obese AT has identified a robust pattern of overexpressed inflammatory genes associated with metabolic disease [12, 13]. Multiple inflammatory mediators abnormally secreted by AT and the crosstalk between immune and metabolic cells can impair insulin signaling and induce oxidative stress and endothelial dysfunction, leading to systemic insulin resistance and cardiovascular disease [5]. Deregulated macrophage-myocyte and macrophage-hepatocyte signaling can impair insulin sensitivity as well [14, 15]. Hypothalamic inflammation, which is induced very rapidly by a high-fat diet [16] may provoke hyperphagia and has been documented to impair insulin release from *β* cells, peripheral insulin action, and potentiate hypertension [17–19]. Thus, chronic excess of nutrients, such as lipids and glucose may simultaneously trigger inflammatory responses, which further disrupt metabolic function, enhancing stress, and inflammation. Such a vicious cycle is identified as a mechanism leading to further metabolic deterioration. Therefore, breaking this vicious circle by resetting the immunological balance in obesity is a crucial approach for the management of MS.

## 3. The Fundamental Role of White Adipose Tissue in Obesity-Induced Inflammation 

Obesity is characterized by excessive expansion of white adipose tissue (AT), which has been thought to be the primary site for the initiation of obesity-associated inflammation. Although AT's principal function deals with energy storage, it serves as an active secretory organ as well. A number of bioactive peptides or proteins, collectively named “adipokines,” are produced and secreted by fat and/or nonfat cells of white AT. They act in an autocrine/paracrine manner to regulate local AT function and also act in an endocrine manner to influence the functions of distant tissues such as liver, skeletal muscle, and cardiovascular and central nervous systems [5, 20].

In obesity, white AT is remodeled dynamically by adipocyte hypertrophy (increased size), hyperplasia (increased number), immune cell infiltration, endothelial cell overactivation, and extracellular matrix overproduction [21–24]. This remodeling may trigger metabolic and hypoxic stress, resulting in activation of multiple inflammatory signaling pathways, ultimately leading to dysregulation of numerous adipokines including proinflammatory cytokines, chemokines, growth factors, acute-phase proteins, and complement-like factors. Virtually, all known adipokines are dysregulated in obesity via multiple mechanisms. Such a deregulation is the main feature of AT low-grade inflammation and contributes to the pathogenesis of MS [5].

## 4. Molecular Mechanisms Mediating White Adipose Tissue Inflammation

Several prominent mechanisms have been highlighted that coordinately mediate white AT inflammation.

*Hypoxia.* In obesity, the growing AT is submitted to hypoxia induced by hypoperfusion at the earliest stages of expansion. Observations have proved that AT is poorly oxygenated in the obese state in humans and rodents due to a reduction in adipose tissue blood flow [25, 26]. This hypoxia could be a determinant mediator of obesity-induced inflammation in AT by activating multiple signaling pathways such as Hypoxia Inducible Factor-1*α* (HIF-1*α*) and Nuclear Factor kappa B (NF-*κ*B) in adipocytes and macrophages, thereby altering the expression of many proinflammatory adipokines [27–29]. In addition, some studies reported that hypoxia also induces AT inflammatory responses indirectly by causing adipocyte death (e.g., apoptosis and necrosis) and lipolysis [30–32].

*Endoplasmic Reticulum Stress.* The endoplasmic reticulum (ER) is responsible for much of a cell's protein synthesis and folding, but it has also a role in sensing cellular stress [33]. Obesity results in conditions that increase the demand on the ER, such as hypoxia and excess of cytokines, lipids, or glucose. Subsequently, the ER stress induces a complex response known as the unfolded protein response (UPR), which alters a cell's transcriptional and translational programs to cope with these stressful conditions and resolve the protein-folding defect [33]. ER stress and the UPR lead to obesity-induced inflammation and metabolic abnormalities by several distinct mechanisms, including the activation of JNK-AP1 (Jun N-terminal Kinase-Activator Protein 1) and IKK (I*κ*B kinase)-NF*κ*B pathways, the induction of the acute-phase response, and the production of reactive oxygen species (ROS) [34, 35].

*Lipotoxicity.* Obesity is characterized by a positive energy balance, and the classical response to this nutrient oversupply is AT hypertrophy. However, the capacity for lipid storage in hypertrophied subcutaneous AT is limited. This limited storage capacity, coupled with the overstimulation of hormone-sensitive lipase, leads to massive increase in free fatty acid (FFA) release and accumulation ectopically. FFAs are potent ligands that activate toll-like receptor (TLR) signaling [36]. TLRs are a family of pattern-recognition receptors that play a critical role in the innate immune system by activating proinflammatory signaling pathways in response to microbial pathogens. AT expresses a broad spectrum of TLRs [37–39]. TLR expression in AT is inducible by inflammatory stimulation and linked to downstream activation of NF-*κ*B or JNK and subsequent release of proinflammatory adipokines [37, 38, 40]. TLR4-deficient mice and C3H/HeJ mice (which have a loss-of-function mutation in TLR4) are partially protected from fat-induced inflammation and insulin resistance in their visceral AT [41, 42]. FFAs also increase the infiltration and activation of macrophages, especially the CD11c+ subset, thereby exacerbating their proinflammatory activity [43].

*Metabolic Endotoxemia.* High-fat diet given to mice chronically increased plasma Lipopolysaccharide (LPS) concentration two to three times; a threshold that has been defined as metabolic endotoxemia [44]. Obese subjects with type 2 diabetes have 76% higher circulating LPS than healthy controls [39] and this high level of LPS decreased significantly after surgical weight loss [45]. Elevated plasma LPS levels result from increased absorption of LPS across the intestinal barrier triggered by high-fat diet. Recently, three underlying mechanisms have been proposed, including the changes in gut microbiota environment [44], the increased availability of chylomicrons [46], and the permeability of the gut epithelium [47–49]. In murine adipocytes, LPS initiates inflammation via TLR4 and induces the secretion of proinflammatory cytokines via downstream activation of NF-*κ*B or mitogen-activated protein kinases (MAPK) signaling pathways [50]. In human adipocytes, stimulation by LPS increases release of Tumor Necrosis Factor-alpha (TNF-*α*), Interleukin-6 (IL-6), and Monocyte Chemoattractant Protein-1 (MCP-1) by NF-*κ*B activation and upregulates TLR2 expression. Newly expressed TLR2 further amplifies proinflammatory signals in AT [39].

## 5. microRNAs as a New Mechanism Mediating White Adipose Tissue Inflammation

microRNAs (miRNAs) are endogenous ~22 nt RNAs that can bind to the 3′-untranslated region (3′-UTR) of target mRNAs to repress mRNA expression at the posttranscriptional level. As a group, miRNAs may directly regulate expression of over 30% of human and mouse genes and more than 60% of human protein-coding genes have been under selective pressure to maintain pairing to miRNAs [51]. Specific miRNAs have been implicated in adipocyte differentiation and mature adipocyte function, including lipolysis, glucose-uptake, and insulin sensitivity [8, 10]. On the other hand, miRNAs have been defined as important immunomodulators by regulating the differentiation, induction, and function of immune cells and the expression of multiple cytokines in the immune system. These findings could shed light on the possible links between miRNAs and adipose tissue immunity/inflammation.

### 5.1. Role of microRNAs in Inflammation

*Role of microRNAs in Adaptive Immunity.* Studies conducted by some groups have demonstrated that miRNA regulation is indispensable for stable immune process. miRNAs appear to have a key role in the differentiation of B-cells. Ablation of whole miRNAs by deletion of Argonaute 2 (Ago2) or Dicer impairs pre-B-cell differentiation and the succeeding peripheral B-cell generation [52]. Several individual miRNAs have also been reported to be involved in the B-cell biology. For example, overexpression of miR-17~92 clusters enhances B-cell proliferation and survival [53], while miR-150 profoundly impairs early B-cell differentiation and mature B-cell responses [54].

miRNAs have been shown to be key regulators of the T-cell lineage induction pathways. Deletion of Dicer at an early stage of T-cell development compromised the survival of T-cell lineage [55]. The best evidence for miRNAs playing a role in specific developmental stages of T-cell differentiation is from miR-181, which reduces the number of T-cells in haematopoietic overexpression systems [56] and also increases the sensitivity of T-cell receptor signaling [57].

In addition, miRNAs also play pivotal roles in the induction, function, and maintenance of the regulatory T-cell (Treg) lineage. miRNAs can enhance thymic and peripheral induction of Treg cells [55, 58]. Dicer-deficient Treg cells almost lost their suppressive capacity and anergic profile [58].

*Role of microRNAs in Innate Immunity.* Several miRNAs (including miR-155, miR-146a, miR-21, and miR-9) have been consistently found to be rapidly induced by innate immune activation (e.g., toll-like receptor), indicating that they may regulate the innate immune response [59]. Target prediction analyses indicate that up to a half of innate immune genes could be under the direct regulation of miRNAs. A study on 613 genes, which regulate immunity utilizing a computational approach, identified 285 genes as miRNA targets. Major targets include transcription factors, cofactors, and chromatin modifiers whereas upstream factors, such as ligands and receptors (cytokines and chemokines) were, in general, poor or nontargets [60].

The mechanisms by which miRNAs regulate cytokine expression include direct regulation by binding to seeding sites in mRNA 3′-UTR and indirect regulation. Computational analyses predict that most cytokines lack miRNA target sites in 3′-UTRs [60]. For example, of the interleukin genes (IL1-29) studied, 9 had predicted miRNA binding sites. Out of 20 interleukin receptor genes examined, only 2 had high probability miRNA target sites. These findings suggest that the regulation of cytokine genes by miRNAs occurs often via indirect mechanism. Recent studies indicate indeed that miRNAs could indirectly regulate cytokine genes via AU-rich elements (ARE) located in the mRNA 3′-UTR by targeting ARE-binding proteins. ARE are the cis-acting structural RNA motifs that are important determinants of cytokine message stability. ARE-mediated mRNA degradation is regulated by a number of trans-acting factors, called the ARE-binding proteins (which include tristetraprolin (TTP), AU rich binding factor 1 (AUF1), and members of the Hu protein R (HUR) family) [61]. These ARE-binding proteins are heavily predicted targets of miRNAs [62]. Repression of several ARE components by miRNAs may alter the levels of inflammatory cytokines as well as of other immune genes [60, 62].

### 5.2. Role of microRNAs in White Adipose Tissue Inflammation

*Obesity-Induced Inflammation Is Associated with Dysregulated Expression of miRNAs in White Adipose Tissue.* Low-grade inflammation in AT is a key feature of obesity. A number of miRNAs have been found dysregulated in white AT during obesity and closely associated with obesity-related metabolic disorders. A recent study identified 21 miRNAs, which were differentially expressed in epididymal AT between lean mice fed a standard diet and mice rendered obese by a high-fat diet [63]. Ortega et al. performed miRNA array on human subcutaneous AT: 50 of the 799 miRNAs tested (6.2%) significantly differed between obese (*n* = 9) and lean (*n* = 6) subjects [64]. Among these 50 miRNAs, 17 were highly correlated with BMI and metabolic parameters (fasting glucose and/or triglycerides) [64]. These data are concordant with those obtained in overweight or obese subjects by Klöting et al. They showed significant correlations between the expression of selected miRNAs and both AT morphology and key metabolic parameters, including visceral fat area, HbA1c, fasting plasma glucose, and circulating leptin, adiponectin, and IL-6 [65]. Another two miRNAs (miR-17–5p and miR-132) were significantly decreased in the omental fat and the circulation of obese subjects [66]. A very recent study defined a set of eleven adipocyte-specific miRNAs downregulated in obese subjects as all of them were concomitantly altered in both the white AT and the isolated adipocytes [67]. Moreover, inflammation* per se* could alter white AT miRNA levels. Xie et al. showed that TNF-*α* treatment of 3T3-L1 adipocytes mimicked the changes of miRNA expression observed in AT of obese mice [68]. With obesity and inflammation being so intrinsically associated, the dysregulated expression of miRNAs in inflammatory adipocytes highly suggests the particular importance of miRNAs in obesity-induced inflammation.

**Figure 1:**
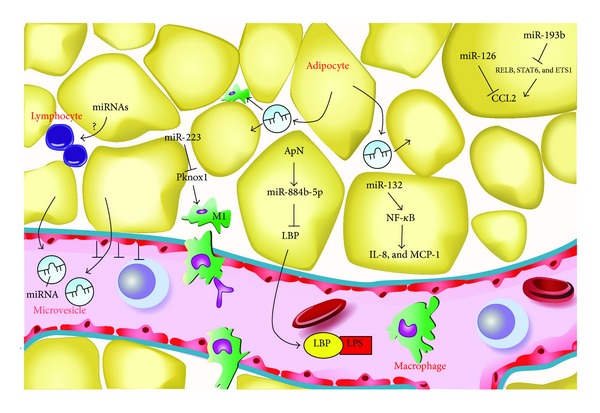
Scheme for the miRNA-mediated mechanisms in regulating white adipose tissue inflammation. The miR-132 activates NF-*κ*B and the transcription of downstream adipokines IL-8 and MCP-1. The miR-223 played a crucial role in modulating macrophage polarization, which suppresses the infiltration of proinflammatory M1 “classically activated” macrophages by targeting Pknox1. The miR883b-5p, which is upregulated by ApN represses the expression and secretion of LBP, a LPS facilitator. The miR-126 reduces the production of CCL2 by targeting directly the 3′-UTR of CCL2, while miR-193b inhibited indirectly the CCL2 production through downregulating the transcription factors of CCL2 (RELB, STAT6, and ETS1). The microvesicles released by adipocytes may either go into circulation or transfer into the neighboring adipocytes or macrophages, probably acting as inflammatory communicators between adipocytes, macrophages, and distant cells. The arrows indicate stimulation or activation; blunted arrows indicate inhibition. ApN, adiponectin; LBP, LPS-binding protein; LPS, lipopolysaccharide; CCL2, chemokine (C-C motif) ligand 2, adapted from [5].

*Individual miRNAs Regulate White Adipose Tissue Inflammation.* Indeed, a few individual miRNAs have been reported as playing a crucial role in the inflammatory state of AT so far (Figure 1). Expression of miR-221 and miR-222 has been positively correlated to TNF-*α* and negatively correlated to ApN expression in white AT of mice [69]. Yet, the direct effects of both miRNAs on these pro- or anti-inflammatory adipokines are still unraveled. miR-132, which was downregulated in human obese omental fat [66], has been reported to activate NF-*κ*B and the transcription of IL-8 and MCP-1 in primary human preadipocytes and in* in vitro* differentiated adipocytes [70]. Zhuang et al. demonstrated that miR-223 played a crucial role in modulating macrophage polarization in a pattern that protects mice from diet-induced AT inflammation and systemic insulin resistance. Consequently, miR-223 suppressed the infiltration of proinflammatory M1 “classically activated” macrophages by targeting Pknox1* in vitro*, while miR-223 deficient mice fed a high-fat diet exhibited increased AT inflammation characterized by enhanced proinflammatory activation of macrophages [71]. The group of Brichard has identified several miRNAs, which were regulated by adiponectin (a promising anti-inflammatory adipokine) in white AT* in vivo*. The miR883b-5p, which was upregulated by adiponectin and downregulated in human obesity repressed the LPS-binding protein (LBP) and TLR4 signaling, acting therefore as a major mediator of the anti-inflammatory action of ApN. Moreover, miR883b-silencing in the* de novo* AT formed from* in vivo* differentiation of preadipocytes also induced LBP production and tissue inflammation [72]. Very recently, Arner et al. overexpressed individual miRNAs (which have been defined downregulated in AT in human obesity) in human adipocytes differentiated* in vitro*. They found that a set of nine miRNAs significantly reduced the secretion of chemokine (C-C motif) ligand 2 (CCL2) which is an initiator of AT inflammation by attracting the migration of inflammatory cells into the tissue. Among these miRNAs, only miR-126 was predicted by* in silico* analysis and confirmed by luciferase reporter assay in 3T3-L1 cells to bind directly to the 3′-UTR of CCL2, whereas miR-193b affected indirectly the CCL2 production through downregulating the transcription factors (TFs) of CCL2 (RELB, STAT6, and ETS1) [67]. Thus, miRNAs may mediate AT inflammation by regulating either the activation of macrophages or the production of adipokines. The mechanism by which miRNAs regulate adipokines in AT seems via direct and indirect mechanisms. miRNAs may act directly on the target inflammatory adipokines or indirectly by first regulating the intermediate machinery components like the TFs, which, in turn, control the expression of adipokines.

In addition to the innate immunity, recent studies have disclosed the importance of the adaptive immunity in AT inflammation. The subsets of the lymphocyte lineage, including CD8+ and CD4+ T-cells, Tregs, Natural killer T-cells (NKT), and B-cells have been revealed to infiltrate into AT which may orchestrate AT inflammation. Lymphocyte infiltration into AT could even precede macrophage infiltration [73, 74]. Yet, the mechanisms underlying the infiltration of lymphocytes into AT remain largely unknown (Figure 1). Whether miRNAs could play roles in modulating the infiltration or activation of the lymphocytes in AT may arouse research interest and requires to be investigated in the future.

*miRNAs Mediate Intercellular Communication.* More recently, adipocytes have been reported to secrete miRNAs in the form of adipocyte-derived microvesicles (ADMs), which may regulate the function of distant or neighboring cells. One study found that microvesicles released from cultured 3T3-L1 adipocytes harbored 143 miRNAs, most of them being adipocyte-specific and reflecting the abundance of their expression levels in the donor cells. Interestingly, the miRNAs-containing microvesicles were transported into cultured macrophages which were incubated with ADMs containing-medium for 24 h; they were also present in rat serum* in vivo* [75]. Yet, whether the miRNAs inside the vesicles were functional in their new location remains to be determined. Furthermore, another group discovered that miRNAs-microvesicle complexes derived from the larger primary rat adipocytes were transferred into and expressed in the smaller adipocytes and were involved in the transcription of multiple genes for lipid synthesis and cell growth [76]. Thus, miRNAs could also be important mediators of cellular crosstalk. Since macrophages and hypertrophied adipocytes are the main sources of AT inflammation, the miRNA-mediated interactions (adipocytes-macrophages and larger adipocytes-smaller adipocytes) may be novel mechanisms for miRNAs contributing to AT inflammation (Figure 1).

## 6. Therapeutic Potential of microRNAs in Metabolic Syndrome

The facts that miRNAs are largely dysregulated in obesity and that specific miRNAs regulate obesity-associated inflammation suggests the potential of these molecules as targets for therapeutic intervention in obesity and obesity-related metabolic disorders.

### 6.1. microRNAs as Diagnostic Tools

Recent studies have demonstrated that miRNAs can be secreted into the circulation. Chen et al. screened serum miRNAs of healthy Chinese subjects and found over 100 and 91 serum miRNAs in male and female subjects, respectively. Yet, how these miRNAs make their way into the circulation is still mysterious [77]. New studies have revealed that circulating miRNAs can reside in microvesicles (exosomes, microparticles, and apoptotic bodies) and in protein/lipoprotein complexes (high density lipoprotein (HDL) and Argonaute 2); these formations make the circulating miRNAs protected and resistant to RNase activity and degradation. Therefore, the levels of miRNAs in serum are remarkable, stable, reproducible, and tissue-specific among individuals. This raises the potential of miRNAs as novel biomarkers for many disorders. Multiple reports have noted the potential use of blood miRNAs as biomarkers for the obesity, cardiovascular diseases, atherosclerosis, and T2DM. Heneghan et al. showed that circulating levels of miR-17-5p and miR-132 were significantly decreased in obese individuals compared to nonobese individuals and reflected miRNA expression in omental fat. The miRNA expression in blood and omental fat from obese patients correlated significantly with BMI, fasting blood glucose, and glycated hemoglobin [66]. Cardiac specific miR-1, miR-208, and miR-499 were induced consistently in blood of patients with myocardial infarction and strongly correlated to markers of cardiac damage including troponin T and creatine kinase-MB (CK-MB) activity [78–81]. Circulating miRNAs have also been proposed as highly sensitive biomarkers for T2DM. In the Bruneck cohort of 822 subjects, decreased serum levels of miR-126 in T2DM emerged as a significant predictor of T2DM and correlated negatively with increasing glucose intolerance [82].

### 6.2. microRNAs as Therapeutic Tools

“Silencing” of key miRNA and “replacement” of certain tissue-specific miRNA whose expression is known to be decreased are potential therapeutic interventions.

*Techniques.* Several techniques have been set up to use the therapeutic potential of miRNAs. miRNAs can be deactivated and silenced by anti-miRNA oligonucleotides (AMOs), “miRNA sponges,” and “miRNA masking” [83]. AMOs are synthetic antisense oligonucleotides that competitively inhibit the interaction between miRNAs and their mRNA targets. The most widely employed types of AMOs are 2′-O-methyl AMO, 2′-O-methoxyethyl AMOs, and Locked Nucleic Acids (LNAs) [84]. Given that miRNAs have been observed to function often in clusters in pathological processes, the “miRNA sponges” have been developed to knockdown multiple miRNAs. This technique requires a vector encoding large quantities of transcripts (anti-miRNAs) which display numerous and tandem binding sites for a cluster of miRNAs of interest. The “miRNA masking” is an alternative miRNA-knockdown strategy to the AMO approach, with the advantage of targeting miRNAs in a gene-specific manner. Unlike AMO, which binds to the endogenous miRNA directly, a miRNA mask binds to the miRNA's binding site located in the 3′-UTR of its target mRNA, thereby avoiding off-target effects [83].

Conversely, with regard to those miRNAs with decreased expression in disease, the fundamental principle strategy is to restore their expression. This can be achieved through miRNA mimicry or plasmid/viral vector-encoded miRNA replacement. miRNA mimics are small chemically altered double-stranded RNA molecules that imitate endogenous miRNAs [83]. Plasmid/viral vectors encoding miRNAs are encouraging strategies to replace miRNA* in vivo*, with good transduction efficiency and minimal toxicity [85, 86].

*microRNAs and Therapeutics.* Since the obesity-induced inflammation is considered to build the common soil for the pathogenesis of MS, some potential anti-inflammatory therapies have been investigated. Several drugs in current clinical practice show the anti-inflammatory properties: the peroxisome proliferator-activated receptor (PPAR) family and nonacetylated salicylates. Other strategies have been explored by targeting the cytokines and chemokines or their receptors (e.g., the anti-TNF or anti-CCR2 (chemokine (C–C motif) receptor 2) therapies) [2]. Although there have been some encouraging results, it is likely that the benefits of these approaches are limited [87, 88]. Therefore, the development of miRNA-based therapeutics has raised interest in this field.

To date, the greatest efforts have been made in exploring the potential application of miRNAs therapeutics in cancer and liver disorders. Gain or loss of function of individual miRNAs have been reported in almost every solid and hematological cancer, with therapeutic suppressing effect in tumor cell proliferation, progression of tumors, and the metastatic process [83]. The liver specific miR-122 has been proved to regulate cholesterol biosynthesis and hepatitis C virus replication. Silencing of miR-122 by intraperitoneal administration of high affinity LNA anti-miR-122 has resulted in dose-dependent lowering of plasma cholesterol in mice and nonhuman primates (monkeys). This was achieved without significant adverse reactions or hepatic toxicity [89, 90].

Likewise, induction or reduction of miRNA expression that is known to be under/upper-expressed and involved in AT inflammation using viral or liposomal delivery of tissue-specific miRNAs to affected AT could potentially result in restoration of immune balance to the AT. Yet, the application of miRNAs therapeutics in AT* in vivo* is poorly investigated because fat cells are insusceptible to selective transfer of exogenous nucleotide due to the containing lipid droplets of adipocytes. Encouragingly, the discovery that miRNAs could be secreted by adipocytes and accepted by neighboring adipocytes or macrophages in the form of adipocyte-derived microvesicles [75, 76] raises the hope that it could be possible to import the targeting miRNAs into AT* in vivo *by the manner of microvesicles. This manner could construct the microvesicles containing specific miRNAs and inject the constructed microvesicles into white AT.

### 6.3. Potential Challenges in microRNA-Based Therapy

Although significant advances have been made in miRNA-based therapy, various challenges remain to be overcome before clinical use. First, one individual miRNA may have multiple potential targets, which may coordinate or antagonize each other's functions. In addition, miRNA-target interactions depend not only on the sequence of the target site but also on the cellular context, in which the interactions occur. This complexity explains the difficulties in predicting the spectrum of side effects and toxicity profiles, which may be associated with miRNA-based therapeutics. Second, replacement of miRNA may potentially lead to oversaturation of endogenous miRNAs pathway. High levels of exogenous miRNA can compete with the endogenous miRNA biogenesis processing, leading to cell toxicity [91, 92]. Another critical issue is the site-specific, safe, effective, and repeated delivery of miRNA* in vivo*. The systemic intravenous delivery of viral or liposomal-mediated miRNA oligonucleotides have been well achieved in cancer and liver disease. However, targeting certain miRNAs in other specific tissues is poorly achieved due to the invasive nature of access for repeat delivery and lack of cell-specific up-take. Restoration of tissue-specific miRNA expression could be a more rational approach for managing local abnormalities with more efficiency and precision and with less systemic side effects [83, 92].

## 7. Conclusion

Evidence has provided that miRNAs are largely dysregulated in white AT in human obesity; specific miRNAs regulate the polarization of macrophages and the expression and secretion of adipokines in white AT; a subset of miRNAs could be packaged into adipocyte-derived microvesicles and delivered into blood or neighboring cells, mediating intercellular communication. Several reports strongly support the roles of miRNAs as important mediators in AT inflammation. Furthermore, with the recent discovery that miRNAs are secreted into circulation in a stable manner and correlated closely with crucial metabolic parameters, the plasma miRNA profiles could therefore be used as attractive biomarkers for the MS. Finally, the establishment of miRNAs-based techniques allows exploring further fascinating roles of miRNAs and their applications in the management of MS. Despite huge challenges lying in miRNA-targeting therapeutics, especially in developing safe and effective delivery of targeting miRNAs in specific tissues* in vivo*, the development of miRNA-based therapeutics for MS is warranted because of the severe problem that metabolic disease poses to society.
